# The Originator of Pathological Anatomy

**Published:** 1871-02

**Authors:** 


THE ORIGINATOR OF PATHOLOGICAL ANATOMY.—About three centuries before the Chistian era, Herophilus, a native of Chalcedon, and Erasistratus, of Iulis, a grandson of Aristotle, laid the foundation-stones of the science of human anatomy. Herophilus, it has been said, resorted to human vivisections, more particularly of criminals. He was the originator of pathological anatomy, and was the first to propose *post mortem* examinations to learn the cause of death. Fallopius, centuries after, denominated him "the evangelist of anatomists."—*Dr. Gouvr. M. Smith's Discourse*

